# Immunotherapeutic Approaches to Peanut Allergy Treatment—Pre-Clinical and Clinical Studies: A Comprehensive Review

**DOI:** 10.3390/jcm14061902

**Published:** 2025-03-12

**Authors:** Kiara Gunawardhana, Petros Martin Raygoza, Catherine Yang, Eslam Mohamed

**Affiliations:** 1 College of Graduate Studies, California Northstate University, Elk Grove, CA 95757, USA; kiara.gunawardhana7410@cnsu.edu (K.G.); petros.raygoza@cnsu.edu (P.M.R.); catherine.yang@cnsu.edu (C.Y.); 2 College of Medicine, California Northstate University, Elk Grove, CA 95757, USA

**Keywords:** allergy, IgE, tolerance, monoclonal antibody, adoptive cell therapy, vaccine, clinical trial

## Abstract

Peanut allergy is a prevalent and potentially life-threatening condition affecting millions of people worldwide, necessitating strict dietary vigilance. Despite its widespread impact, current treatment options are predominantly limited to allergen avoidance and emergency management of allergic reactions. This review explores contemporary immunotherapeutic strategies aimed at achieving long-term relief for individuals with peanut allergy. We conducted a comprehensive literature review to discuss different treatment approaches, such as subcutaneous immunotherapy (SCIT), epicutaneous immunotherapy (EPIT), oral immunotherapy (OIT), and sublingual immunotherapy (SLIT), focusing on their mechanisms, efficacy, and safety profiles. Additionally, the review delves into novel approaches such as monoclonal antibodies targeting IgE and other critical immune pathways, adjuvanted therapies utilizing nanoparticles and gut microbiota, and advances in adoptive cell therapy including CAR-T cells and regulatory T cells. Furthermore, we highlight some clinical trials that test the efficacy and safety of these novel immunotherapeutic approaches in patients with peanut allergy. Collectively, we provide an overview of advancements in immunotherapeutic interventions for peanut allergy and recommendations for personalized immunotherapy regimens, ultimately paving the way for more effective treatment strategies.

## 1. Introduction

**Figure 1:**
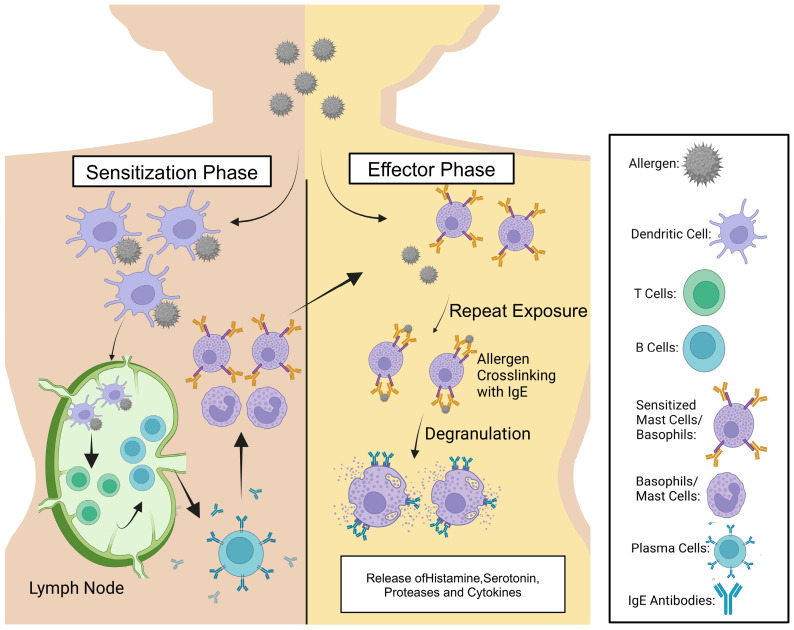
Allergic disease development: The figure above is a schematic representation of the sensitization and effector stages in allergy development. Additionally, the diagram illustrates the roles of various immune cells and mediators involved in these stages.

Peanut allergy (PA) is a prevalent and challenging childhood IgE-mediated condition that often persists into adulthood, historically lacking effective treatment options. For individuals with peanut allergy, the consumption of trace quantities of peanuts or peanut products can provoke fatal anaphylaxis [1]. Allergic responses are initiated when atopic individuals produce IgE antibodies in response to allergens, which are typically proteins or glycoproteins, in this case, specific proteins found in peanuts. Individuals who do not possess an allergy produce IgM, IgG, or IgA when encountering the allergen without producing any symptoms. However, in allergic individuals, the first exposure to the allergen activates T helper 2 (Th2) cells, which leads to the production of cytokines that stimulate IgE assembly and release. The IgE antibodies will then attach themselves to the Fc epsilon receptors (FcεRs) on hematopoietic cells such as mast cells and basophils, thereby sensitizing them to the allergen. When the individual is re-exposed to the allergen, the allergen crosslinks surface IgE molecules on mast cells in tissues or circulating basophils, leading these cells to undergo degranulation [2]. The degranulation process results in the release of preformed and newly synthesized mediators, such as histamine, leukotrienes, and cytokines, leading to vasodilation, heightened vascular permeability, and inflammation, thereby manifesting as allergic symptoms (Figure 1). In addition to the biochemical signals triggered by those mediators, mechanical forces within the allergy microenvironment play a crucial role in modulating immune responses. The mechanical forces exerted by IgE crosslinking on mast cells, molecular forces generated by actin cytoskeleton and microtubules, the degree of stiffness of extracellular matrix (ECM) components are examples of mechanical forces involved in type I hypersensitivity disorders. These forces activate mechanosensitive signaling pathways including Mitogen-activated Protein Kinase (MAPK) and Rho GTPases that influence immune activation in allergies. Understanding the mechanobiology of allergy opened new frontiers for therapeutic interventions [3].

Peanut allergy affects around 2% of the population in western nations and the prevalence has been increasing globally. Unlike other food allergies such as egg, milk and soy, which are outgrown, peanut allergy remains persistent through adulthood in 75–80% of children [4]. The imperative to identify efficacious therapies for peanut allergy stemmed from its high prevalence together with its overall economic impact [5,6]. Because sufficient reliable therapies were not available until recently, peanut allergy management often relied on avoidance of the allergen, although adverse events would often occur in patients due to accidental ingestion [7]. However, over recent years, there have been several immune-based interventions that have shown therapeutic benefits in the treatment of peanut allergy, including antigen-based immunotherapy and non-antigen-based immunotherapy. In this review, we will be discussing several immunotherapeutic approaches in addition to their mechanisms of action for the treatment of peanut allergy and their efficacy in both preclinical and clinical settings

## 2. Antigen-Based Therapy

Antigen-based therapy for peanut allergy is a category of immunotherapeutic approaches that involve administering increasing doses of a particular antigen, in this case, the peanut allergen, to induce desensitization or immune tolerance. A synthetic construct of multiple antigens or epitopes offers an innovative and more comprehensive approach to manage hypersensitivity. This scaffold design allows for the interaction with multiple targets, thus inducing a broader spectrum of immune tolerance [8]. Antigen-specific immunotherapy (AIT) includes a variety of subcategories based on the route of administration (Table 1), including subcutaneous immunotherapy (SCIT), epicutaneous immunotherapy (EPIT), oral immunotherapy (OIT), and sublingual immunotherapy (SLIT). Desensitization to an allergen occurs when there is a rise in the threshold dose of the food allergen that is needed to cause an allergic reaction in the individual following repeated exposure [9]. AIT aims to induce immune tolerance in individuals by changing their immune response to the posed antigen. With AIT, gradually increasing antigen doses will allow the immune response to shift towards tolerance by suppressing Th2 responses, encouraging regulatory T cells, and adjusting allergic inflammation until the immune response is decreased [10].

T lymphocytes, particularly regulatory T cells (Tregs), play a pivotal role in establishing tolerance to food allergens. While Th2 cells drive allergic reactions, Tregs promote immune tolerance. These cells secrete regulatory cytokines like interleukin-10 (IL-10) and transforming growth factor-beta (TGF-β), and express specific surface markers, including CD25, which collectively modulate immune responses. Experimental studies utilizing foxp3 knockout mouse models have demonstrated the development of inflammatory responses, highlighting the crucial role of Tregs in immune regulation. In addition to this, the transfer of Tregs has been shown to suppress allergic reactions in mice models effectively [11]. Clinical observations suggest that children with food allergies often exhibit decreased levels of Tregs, and peanut allergy is most common in younger children [12].

Therapeutic interventions such as oral immunotherapy and administration of low-dose interleukin-2 (IL-2) have been shown to enhance Treg functionality, thereby representing promising strategies for the management of food allergies such as peanut allergy [11]. Th3 cells, which produce TGF-β, contribute to the establishment of oral tolerance. TGF-β is crucial for promoting immune tolerance by promoting Tregs and inhibiting effector T cells, preventing excessive immune responses. It also induces anti-inflammatory cytokines, like IL-10 [13]. Additionally, B cells, including immunosuppressive B regulatory cells (Bregs), are vital for immune responses as they produce antibodies and regulate T-cell activation. Bregs suppress effector T cells through cytokines such as IL-10, TGF-β, and IL-35 in a similar mechanism to Tregs. They also produce IgG4, an antibody that is notable for its role in immune tolerance. IgG4 can be produced in response to exposure to an allergen and blocks IgE-mediated allergic reactions, potentially assisting in tolerance induction. Moreover, Bregs can impede T effector cell proliferation and the maturation of dendritic cells while promoting the production of anti-inflammatory molecules, therefore demonstrating their intricate role in immune regulation in conditions such as peanut allergy [11].

### 2.1. Subcutaneous Immunotherapy (SCIT)

Unlike subcutaneous immunotherapy, which induces tolerance to airborne allergens, subcutaneous injections of food allergens are associated with high rates of severe allergic reactions. A novel subcutaneous immunotherapy (SCIT) using a modified peanut extract (HAL-MPE1) is being developed to treat peanut allergy, with an emphasis on enhancing safety and tolerability. Early clinical trials in adults showed promising results, with 92% of participants achieving the maximum dose, leading to the expansion of studies into adolescent and pediatric populations to further assess its efficacy and safety profile [14]. Compared to other therapies, SCIT has a substantially higher risk of systemic anaphylaxis reactions. Because of its nature, SCIT entails subcutaneous injection of the allergen, which can result in allergens entering the bloodstream. In certain instances, these allergens may be recognized as threats by the immune system, leading to a systemic allergic reaction [15]. Several studies have highlighted the role of adjuvants in improving SCIT by reducing IgE levels and decreasing systemic side effects [16]. Aluminum salts (Alum) are frequently employed as adjuvants in SCIT. SCIT’s mechanism includes activating dendritic cells, presenting antigens, and stimulating Treg cells to suppress the Th2 response, or potentially leading to a Th1 response when combined with an alum [17].

### 2.2. Epicutaneous Immunotherapy (EPIT)

EPIT is a noninvasive strategy for AIT that involves the delivery of antigens to the intact skin. This strategy aims to elicit an immune response while minimizing systemic reactions that may occur due to the poor vascularization of the epidermis. Antigens applied to the skin target epidermal dendritic cells, which migrate to draining lymph nodes after internalizing the antigen, leading to the induction of immune tolerance. EPIT promotes immune tolerance instead of triggering an inflammatory response by targeting specific immune subsets. By routing the antigen through the epidermis, EPIT delivers allergens to pro-tolerogenic Langerhans cells (LCs), fostering immune tolerance. The use of microscale doses of allergens reduces the likelihood of local inflammation, distinguishing EPIT from other therapies [18]. In EPIT for peanut allergy, small quantities of peanut allergens are repetitively administered through an epicutaneous delivery system. Preclinical investigations in mouse models have demonstrated that EPIT can reduce Th2 response, lead to desensitization, and provide protection for subsequent oral exposure to peanuts [19]. Multiple clinical trials had tested the efficacy and tolerability of peanut EPIT among patients with peanut sensitivity, with some participants achieving a significant and promising treatment response [20,21,22,23]. However, peanut desensitization via EPIT has not yet received FDA approval. Challenges impeding its market authorization include limitations in the design of the epicutaneous delivery system, regulatory constraints related to manufacturing processes, and the occurrence of short-term skin reactions observed in clinical trials. Furthermore, the absence of reliable biomarkers to predict treatment response, coupled with the necessity of oral food challenges to assess efficacy, poses significant barriers to its widespread clinical implementation and long-term viability as a standard therapeutic option [24]. Still, EPIT represents a promising treatment method for the safe and effective management of peanut allergy.

### 2.3. Oral Immunotherapy (OIT)

Recent clinical investigations into OIT for peanut allergy have showcased considerable potential, exhibiting high rates of clinical desensitization and a favorable safety profile while administered under the supervision of trained healthcare providers. Utilizing a gradually increasing oral dosing regimen has been shown to be a more efficacious and safer approach compared to a rapid protocol, leading to a reduction in adverse effects. The development of oral tolerance involves the complex interactions between intestinal mucosa tissues and cells. Gut-associated lymphoid tissue (GALT), particularly Peyer’s patches, plays a significant role in this process by sampling allergens and facilitating immune regulation. Specialized M cells within Peyer’s patches transport peanut antigens to antigen-presenting cells, which subsequently promote the differentiation of Tregs, contributing to immune tolerance and the suppression of allergic responses [25]. Dendritic cells transport food antigens from the mucosa to lymph nodes, where they interact with T-cells, promoting the formation of Tregs. These Tregs then migrate back to the mucosa and undergo expansion, which is facilitated by the anti-inflammatory effect of IL-10 produced by CX3CR+ macrophages. Mechanisms that contribute to oral tolerance and the suppression of immune responses involve changes in basophil activation, mast cell reactivity, and anti-inflammatory cytokine production due to the induction of Tregs [26]. The FDA has granted approval for Peanut (Arachis hypogaea) Powder in 2020, marketed under the tradename Palforzia, for the oral immunotherapeutic management of peanut allergy in individuals ages 4–17. This treatment aims to induce immune tolerance to peanut protein by administering doses orally [27]. The FDA approval for Palforzia came as a result of randomized controlled trials that showed the substantial capacity of peanut OIT to induce desensitization in more than half of the children’s population with peanut allergies, elevating their threshold for reaction to a minimum of 10 peanuts [28,29].

### 2.4. Sublingual Immunotherapy (SLIT)

SLIT is an alternative to injection-based immunotherapy for peanut allergy by utilizing the sublingual route to effectively induce antigen-specific tolerance. This method facilitates direct interaction between peanut proteins and local tolerogenic antigen-presenting cells (APC) in the oral mucosa, therefore enhancing the induction of tolerance by bypassing gastric digestion [30]. Peanut SLIT involves the administration of increasing doses of peanut extracts under the tongue over time, with doses starting in micrograms and increasing to 2 mg, which is notably less than the doses used in OIT. A recent clinical trial has demonstrated that peanut SLIT can effectively induce desensitization in young children and provide lasting protection after discontinuation of therapy [31].

## 3. Anti-IgE Monoclonal Antibodies

Monoclonal antibodies targeting IgE represent a promising category of immunotherapeutic interventions for peanut allergy. These antibodies selectively bind to IgE, thereby attenuating its capacity to initiate allergic reactions in individuals afflicted with peanut allergy. The employment of monoclonal antibodies in peanut allergy treatment initially emerged as a viable alternative to antigen-based oral immunotherapy. This shift was prompted by the observation that a notable subset of patients undergoing the latter therapy experienced systemic allergic reactions necessitating epinephrine administration [32]. A monoclonal antibody (mAb) is an antibody generated by a B-cell clone and exhibits a remarkable specificity towards a particular antigen. The design of monoclonal antibodies can be tailored to target specific molecules within the body, including antibody receptors and proteins [33]. This specificity renders them valuable in a range of therapeutic contexts, encompassing the treatment of autoimmune diseases, infections, cancer, and allergies. Bispecific antibodies (BsAbs) are another emerging technology due to their ability to simultaneously target two distinct antigens. Unlike traditional monoclonal antibodies, BsAbs have a dual-binding capacity to modulate multiple immune pathways [8].

Therapeutic strategies such as anti-IgE monoclonal antibody (mAb) therapy are designed to modulate Th2-mediated immunity. This approach involves administering an antibody that impedes the binding of IgE to FcεRI receptors on mast cells and basophils, thereby reducing IgE-mediated hypersensitivity reactions to allergens [9]. In addition to blocking IgE binding, anti-IgE therapy reduces the expression of FcεRI receptors on basophils or mast cells and may interfere with allergen-specific T-cell activation by affecting antigen-presenting cell processing mediated by FcεRI and FcεRII receptors [34]. Omalizumab, or Xolair by trade, a humanized monoclonal anti-IgE antibody, specifically targets an epitope on the CH3 domain of the IgE heavy chain, crucial for its interaction with both high-affinity and low-affinity IgE receptors (FcεRI and FcεRII, respectively) [9].

Multiple clinical studies have underscored the efficacy of Omalizumab in managing allergic rhinitis, asthma, and when used alongside allergen-specific immunotherapy for pollen allergies [9]. However, Omalizumab was not considered a therapeutic intervention in food allergies until recently when the FDA approved Omalizumab for ameliorating allergic reactions in individuals with food allergies, particularly peanut allergies. Omalizumab operates by diminishing circulating serum IgE levels, reducing high-affinity IgE receptor expression, and inhibiting basophil histamine release [32]. The clinical trial leading to FDA approval of Omalizumab for food allergy therapy demonstrated a substantial elevation in the threshold for peanut and other food allergens compared to a placebo effect among participants. Omalizumab doses, based on participant weight and IgE levels, were administered subcutaneously during the trial [35]. In addition to this, a small pilot study combining Omalizumab with peanut OIT in children at high risk of peanut allergy demonstrated successful oral desensitization in most participants, although some experienced moderate to severe adverse events [9]. Furthermore, another study utilizing a humanized monoclonal anti-IgE antibody (TNX-901) showed promise in enhancing peanut tolerance among patients with peanut allergy; however, the immune response varied among individuals [34].

## 4. Adjuvanted Therapies

Adjuvanted therapy in peanut allergy involves the use of adjuvants to potentiate the immune response to peanut allergens. This approach aims to strengthen and prolong the immune reaction, potentially leading to greater efficacy.

### 4.1. Nanoparticles

Nanoparticles, composed of polymers and macromolecules, serve as adjuvants in allergy immunotherapy to enhance the delivery of antigens to the immune system. Nanoparticles are readily internalized by immune cells such as dendritic cells and macrophages, thus facilitating allergen delivery. This offers several advantages, including protection from enzymatic degradation, the controlled release of allergens, and the ability to co-encapsulate allergens with immunopotentiators, which are compounds that directly activate immune cells through specific pathways or receptors [36]. In the realm of parenteral administration, polymers characterized by slow degradation rates, such as poly(lactic-co-glycolic acid (PLGA), are preferred for their capacity to maintain the gradual release of allergens. Additionally, nanoparticle compositions incorporating PLGA, chitosan, or poly(anhydrides) have demonstrated efficacy in conveying allergens via mucosal pathways [37].

For example, CpG-coated PLGA nanoparticles containing peanut extract (CpG/PN-NPs) have been investigated for their ability to safely deliver peanut allergens in a therapeutic context. The incorporation of adjuvants such as CpG is intended to enhance the immune response to peanut allergens, with the ultimate objective of inducing desensitization or tolerance to peanuts over time. In a murine model, CpG/PN-NPs demonstrated improved protection against anaphylactic challenges compared to vehicle-treated mice. Mice treated with nanoparticle adjuvant therapy exhibited a significant decrease in IFN-γ and Th2 cytokines, including IL-4, IL-5, and IL-13 [38].

In another study, oral administration of poly(anhydride) nanoparticles loaded with a peanut protein extract resulted in a balanced Th1- and Th2-mediated antibody response, leading to decreased levels of specific IgE. Additionally, mice treated with these nanoparticles demonstrated reduced concentrations of pro-Th2 cytokines (IL-4, IL-5, IL-6) and elevated levels of IFN-γ and IL-10 compared to the control cohort [39]. Collectively, nanoparticles represent a promising avenue for precise allergen delivery, augmentation of immune responses, and potentially, improving the efficacy and safety profiles in peanut allergy treatments.

### 4.2. Pattern Recognition Receptors—Ligands

As substances that can augment the immune response to an antigen, the ligands for pattern recognition receptors (PRRs) are recognized for their ability to facilitate antigen uptake by antigen-presenting cells (APCs), notably dendritic cells (DCs), and frequently induce APC activation. Toll-like receptors (TLRs), alongside C-type lectin receptors (CLRs) and nucleotide-binding oligomerization domain protein 1 (NOD1), are categorized as PRRs due to their ability to recognize pathogen-associated molecular patterns (PAMPs). PRR-mediated adjuvants have traditionally been associated with enhancing Th1 or Th17 immunity, which is particularly important in the context of food allergies such as peanut allergy as it downregulates the Th2 response commonly found in IgE-mediated allergy [40,41].

### 4.3. Gut Microbiota

Because gut microbiota has a fundamental role in regulating gut immunity, researchers have looked into the potential benefits of probiotics as adjuvants to induce gut immune modulation in joint administration with peanut OIT [42]. Probiotics are live microorganisms that confer a beneficial effect on the host and hold the potential to promote immune tolerance. Through their ability to modulate the gut microbiota and immune responses in the gut, probiotics show promise in preventing and treating food allergies such as peanut allergy, with certain strains demonstrating efficacy in clinical trials. The modulation of the gut microbiome using probiotics has the potential to bolster immune tolerance in food allergies by upregulating anti-inflammatory cytokines such as IL-10, promoting IFN-γ production to mitigate Th2-driven allergic responses, and regulating cytokine production by immune cells [43]. Despite OIT’s success in achieving desensitization in most children, sustained unresponsiveness remains challenging to attain. A groundbreaking study explored a novel approach combining a probiotic (*Lactobacillus rhamnosus* CGMCC) with OIT [44]. Initial results after one year of treatment revealed that 89% of participants in the probiotic-OIT (PPOIT) group achieved desensitization, contrasting with only 7% in the placebo group that was given the peanut protein without an additional probiotic. Moreover, 82% of the PPOIT group achieved sustained unresponsiveness, highlighting the approach’s potential. Subsequent 4-year follow-ups showed that 67% of the PPOIT group could safely consume peanuts, compared to a mere 4% in the placebo group. This study marks the first demonstration of prolonged sustained unresponsiveness with any peanut OIT regimen, underscoring its effectiveness. These findings suggest that the probiotic-OIT approach holds promise for treating peanut allergies offering the prospect of long-term tolerance with individuals potentially being able to consume peanuts infrequently without allergic reactions [44]. In addition, animal studies indicate that probiotics such as *Bifidobacterium infantis*, *Bacillus coagulans*, and *Lactobacillus plantarum* may mitigate intestinal inflammation and symptoms linked to food-induced anaphylaxis. Consequently, the concurrent administration of OIT with these probiotics has demonstrated encouraging outcomes in promoting tolerance to peanut allergy, suggesting a potential synergistic impact [45].

## 5. Adoptive Cell Therapy

Adoptive cell therapy (ACT) may be emerging as a promising approach for addressing peanut allergy. This therapy involves the transfer of cells into a patient, which can be sourced from either the patient or a donor, typically derived from the immune system. In the context of peanut allergy, ACT would aim to modify the immune response to peanuts, with the ultimate goal of reducing or eliminating allergic reactions. ACT has shown remarkable success as an immunotherapeutic approach to treating cancers through the infusion of tumor-infiltrating lymphocytes (TILs). Having shown success in the treatment of cancer, adoptive cell therapies employing naturally occurring allergen-specific regulatory T cells (Tregs) and chimeric antigen receptor (CAR)-T cells can be implemented in the management of IgE-mediated allergy [46]. Through the introduction of immune cells capable of regulating and/or eliminating the activity of other immune cells involved in the allergic response, ACT represents a compelling therapeutic intervention for peanut allergy.

Specific to allergies, Tregs can be infused to regulate the immune response against allergens, such as those responsible for peanut allergy. Tregs are pivotal in maintaining immune tolerance, preventing exaggerated immune reactions to innocuous substances like food proteins. The process involves isolating Tregs from the patient’s blood, expanding them in vitro, and reintroducing them into the patient’s system. This strategy aims to bolster the Treg population, enabling them to dampen the activity of other immune cells participating in the allergic response. FoxP3+ Tregs have shown therapeutic promise in numerous preclinical models and clinical trials. These trials have focused on preventing graft-versus-host disease in organ transplantation and managing various autoimmune diseases, including type 1 diabetes mellitus, inflammatory bowel disease, systemic lupus erythematosus, multiple sclerosis, and allergy [47]. Researchers might consider investigating the administration of Tregs as an innovative therapeutic strategy tailored to peanut proteins. This approach would aim to reprogram the immune system to tolerate these antigens, potentially reducing or eliminating allergic reactions upon future exposure [48].

However, while ACT presents an innovative immunotherapeutic avenue for food allergies, including peanut allergy, it remains highly experimental. The immunomodulatory nature of ACT raises important considerations, such as immune-related toxicities, off-target effects, and the durability of immune tolerance. These risks must be carefully evaluated to ensure both safety and efficacy. Although ACT may offer a potential option for individuals at high risk of life-threatening anaphylaxis, it remains an aggressive strategy requiring extensive clinical validation [49]. Further research is essential to determine its viability in allergic disease management.

CAR-T cells, also known as chimeric antigen receptor T cells, are a novel form of immunotherapy under investigation for the treatment of allergies. This approach involves genetically modifying T cells from the patient’s own immune system to express a chimeric antigen receptor (CAR), as a fusion protein that merges the antigen-binding domain of an antibody with signaling domains sourced from T-cell receptors and additional co-stimulatory molecules. This engineered structure enables the CAR to specifically recognize and bind to a precise antigen located on B cells. Upon encountering a B cell expressing the target antigen, the CAR binds to the antigen, triggering a cascade of signaling events that activate the CAR-T cell, subsequently initiating an immune response against the target B cell. Following activation, the CAR-T cell can eliminate the target B cell through diverse mechanisms, including the release of cytotoxic molecules or the induction of apoptosis. Furthermore, a subset of CAR-T cells can persist in the body as memory cells, thereby offering enduring immunity against B cells expressing the target antigen [50]. The rationale behind employing CAR-T cells in allergy therapy is to eliminate the IgE-producing B cells, thereby mitigating or eradicating IgE-mediated allergic reactions.

A study has explored the application of CAR-T cell therapy in allergic diseases by selectively eliminating B cells responsible for producing IgE, the primary driver of these allergic reactions. Specifically, the CAR-T cells developed for this purpose are designed to target the transmembrane form of IgE (mIgE), which is exclusively present in IgE-secreting B cells. This study emphasized the creation of two distinct CAR designs: EMPD CAR, which recognizes a specific region (EMPD) on mIgE, and the ACED CAR, which targets the alpha chain extracellular domain of the high-affinity IgE receptor FcεRI. Both CAR designs have demonstrated the ability to selectively target cells expressing membrane-bound IgE in preclinical investigations [51]. Compared to Palforzia, an FDA-approved oral immunotherapeutic approach for the treatment of peanut allergy, mIgE-targeted CAR-T cells would be administered one time only and can potentially eliminate disease exacerbations.

Engineered approaches encompass the genetic modification of polyclonal Tregs through the introduction of chimeric antigen receptors (CARs) or predefined T-cell receptors (TCRs), enabling them to recognize particular antigens. This modification results in the generation of a plentiful reservoir of antigen-specific Tregs [44]. In a recent study, researchers utilized a CAR-Tregs approach in an ovalbumin (OVA) allergic model targeting mast cells (specifically IgE-sensitized mast cells) and B cells. These engineered Tregs, termed OVA-BAR Tregs, provided protection against hypothermia in sensitized mice following the OVA challenge [52]. Despite promising results in preclinical models, human clinical trials investigating the use of adoptive cell therapy in allergic inflammation have not yet been conducted. Researchers should further investigate the engineering of CAR-T cells targeted against B cells as an innovative therapeutic strategy for treating peanut allergies since this approach shows potential for providing sustained symptom relief, and possibly a cure for allergy and other inflammatory diseases, through a single administration.

Studies have investigated adoptive cell transfer of allergen-expressing B cells as a potential therapeutic approach for IgE-mediated allergies [53]. One study focused on inducing tolerance in mice using grass pollen allergen Ph1 p 5-expressing immune cells, among which CD19+ B cells emerged as promising candidates for allergen-specific cell therapy. Specifically, transferring Ph1 p 5+ CD19+ B cells to mice followed by their sensitization with the same antigen prevented the development of Ph1 p 5-specific IgE and IgG1 antibody responses for up to 26 weeks. The transfer of Ph1 p 5+ B cells induced sustained tolerance that was specific to Ph1 p 5, as antibodies specific to an unrelated control antigen were still detected. Treated mice exhibited preserved lung function and no Ph1 p 5-induced mast cell degranulation, suggesting the potential of CD19+ B cells as a prophylactic treatment for IgE-mediated allergies [53].

## 6. Clinical Trials

Several ongoing clinical trials are investigating innovative strategies for managing peanut allergy (Table 2). One such trial, NCT01197053, has investigated EPIT with Viaskin^®^, developed by DBV Technologies, as a potentially safer alternative for peanut allergy treatment. EPIT delivers controlled amounts of allergens to the skin’s outer layers, avoiding direct contact with the bloodstream and thereby reducing the risk of systemic reactions. This pilot study aimed to demonstrate the safety and efficacy of EPIT in desensitizing peanut-allergic children, enabling them to tolerate increased amounts of peanut protein without experiencing symptoms.

Another trial, registered as NCT00932282, evaluated a combination therapy approach that involves OIT with Omalizumab treatment. This trial aimed to assess the safety and effectiveness of this combined therapy in reducing the risk of severe allergic reactions and inducing tolerance to peanuts. The treatment consisted of two components: peanut oral immunotherapy (PnOIT) and Omalizumab, with the latter hypothesized to decrease side effects and accelerate the desensitization process. The treatment arms were tested in both a 12-month and 24-month time periods.

NCT01373242 focused on SLIT for peanut allergy, which has shown promise as a safe alternative to reduce allergic reactions during oral food challenges involving peanuts. The primary objective of this study was to desensitize peanut-allergic children using peanut SLIT and evaluate the persistence of the non-reactive state of their immune systems after treatment. Children aged 1–11 years were enrolled after a double-blind, placebo-controlled food challenge (DBPCFC). Following at least 48 months of peanut SLIT, subjects underwent a second DBPCFC to evaluate desensitization. Subjects that were unable to consume more than 300 mg of peanut protein without symptoms discontinued peanut SLIT, while those who consumed more than 300 mg avoided peanuts for a period, followed by a third DBPCFC. If desensitization was maintained, subjects were advised to consume a daily peanut equivalent to sustain the effect.

The 2021 clinical trial, NCT03211247, a Phase III study aimed at assessing the safety and efficacy of Viaskin^®^ Peanut for desensitizing children aged 1 to 3 years with peanut allergies. Utilizing a double-blind, placebo-controlled, randomized design, this trial involved a 12-month treatment period with epicutaneous immunotherapy (EPIT). The primary objective was to determine the effectiveness of Viaskin^®^ Peanut in inducing desensitization within this young age group, addressing a gap in current treatment options for these patients.

NCT03881696 is a clinical trial investigating the use of Omalizumab injections as monoclonal antibody therapy in conjunction with multi-allergen oral immunotherapy (OIT) to improve the ability of individuals with multiple food allergies to consume their allergenic foods. The study was divided into three stages: Stage 1 evaluated whether Omalizumab can reduce allergic reactions to peanut and other food allergens with prolonged use, including an open-label extension for long-term effects assessment. Stage 2 compared a short course of Omalizumab combined with multi-allergen OIT to a longer course of Omalizumab to determine efficacy in reducing allergic reactions. Stage 3 assessed if participants can safely consume peanut and other allergenic foods in their regular form after discontinuing both treatments. The study also evaluated the safety and effectiveness of the treatments and their impact on the immune system.

The POSEIDON study, registered as NCT03736447, is a Phase III clinical trial designed to assess the efficacy and safety of AR101, an oral immunotherapy, in young children with peanut allergies. This trial employed a randomized, double-blind, placebo-controlled methodology and is conducted at 23 study sites. The study utilized the FDA-approved oral immunotherapy regimen Palforzia, which is typically indicated for individuals aged 4–17 with peanut allergies, but in this trial, the focus was on peanut-allergic children aged 1 to under 4 years. The Characterized Oral Desensitization Immunotherapy (CODIT™) regimen is employed to evaluate whether AR101 can achieve effective desensitization in this younger cohort compared to a placebo.

A recent study that is still recruiting, NCT06297083, examines three treatment approaches for peanut allergy in a randomized trial involving 130 children aged 1 to 10 years with confirmed peanut allergy. The treatments include high-dose rapid escalation peanut oral immunotherapy combined with probiotic (HD PPOIT), high-dose rapid escalation peanut oral immunotherapy combined with probiotic placebo (HD OIT), and low-dose slow escalation peanut oral immunotherapy combined with probiotic placebo (LD OIT). However, this study was withdrawn due to budget and timeline compliance.

In addition to probiotics, fecal microbiota transplantation (FMT) has been another promising avenue of changing the gut microbiome to treat allergies. This process involves the transfer of microbial communities, for example, from healthy donors to recipients with gut dysbiosis. One clinical trial that tests FMT in food allergies aims to evaluate the safety and efficacy of oral-encapsulated FMT for patients with peanut allergy (NCT02960074). Patients in this study will either undergo FMT alone or FMT with an antibiotic pre-treatment. Once this precondition is completed, patients will undergo a double-blind placebo-controlled food challenge with peanut protein [54].

## 7. Limitations, Future Directions, and Recommendations

Despite advancements in various immunotherapeutic approaches, significant knowledge gaps remain, hindering their efficacy and application. The precise immune mechanisms driving desensitization are not fully understood, and long-term efficacy and safety data are limited, leaving uncertainty about the duration of desensitization and potential long-term adverse effects. There is significant variability in patient responses, underscoring the need to explore genetic, epigenetic, and environmental influences. While the FDA-approved treatment protocols exist, optimal dosing regimens for different age groups are not well established, necessitating research to identify the most effective approaches. The potential for combining antigen-based therapies with other treatments, such as adjuvants or probiotics, remains underexplored. Additionally, the mechanisms behind adverse reactions resulting from the treatment are not well understood, warranting further investigation. Addressing these gaps through rigorous research and clinical trials is essential for advancing immunotherapeutic approaches for peanut allergy and achieving consistent, reliable results for patients.

Future directions for immunotherapeutic approaches to peanut allergy should focus on exploring the precise immune mechanisms underlying desensitization. This would enable the development of more targeted and effective therapies. Extensive long-term studies are required to confirm the sustained efficacy and safety of these treatments, addressing concerns regarding the duration of desensitization and potential long-term adverse effects. To optimize dosing regimens and treatment protocols across different age groups, extensive research is required as current guidelines remain inadequate. Researchers should invest in exploring combination therapies between adjuvants and other existing therapies such as monoclonal antibodies or antigen-based approaches to cultivate positive outcomes. Additionally, adoptive cell therapy has proven effective in the treatment of asthma, an IgE-mediated condition, which may indicate its success as a therapeutic approach for the treatment of peanut allergy. Elucidating the mechanisms underlying adverse reactions and non-responsiveness will contribute to the refinement of treatment strategies, thereby mitigating risks and enhancing patient safety. Progress in these domains, facilitated by rigorous clinical trials and interdisciplinary research, will be pivotal for the development of next-generation immunotherapies for peanut allergy.

Personalized medicine approaches are essential for tailoring treatments to individual patient profiles, thereby enhancing efficacy and minimizing variability in responses. A personalized approach to peanut allergy immunotherapy necessitates the customization of treatments based on individual patient characteristics and responses. Antigen-based therapy can be individualized by employing diagnostics to identify the specific peanut proteins responsible for allergic reactions, as well as adjusting dosages based on individual tolerance levels, thereby facilitating targeted desensitization protocols. Adjuvanted therapy can be modified by selecting adjuvants that enhance immune tolerance according to a patient’s immune profile, potentially incorporating cytokine or toll-like receptor agonists to optimize the immune response. Monoclonal antibodies, such as Omalizumab, can be utilized to neutralize IgE antibodies, with dosages adjusted based on genetic markers predictive of responsiveness and a patient’s IgE levels. Adoptive cell therapy involves engineering the patient’s own T cells to enhance regulatory functions and suppress allergic reactions, with the selection and modification of T cells guided by the individual’s specific immunological and genetic makeup. By integrating these personalized strategies, immunotherapy for peanut allergy can achieve greater efficacy and safety, thereby minimizing the risk of adverse effects and improving patient outcomes.

## 8. Conclusions

**Figure 2:**
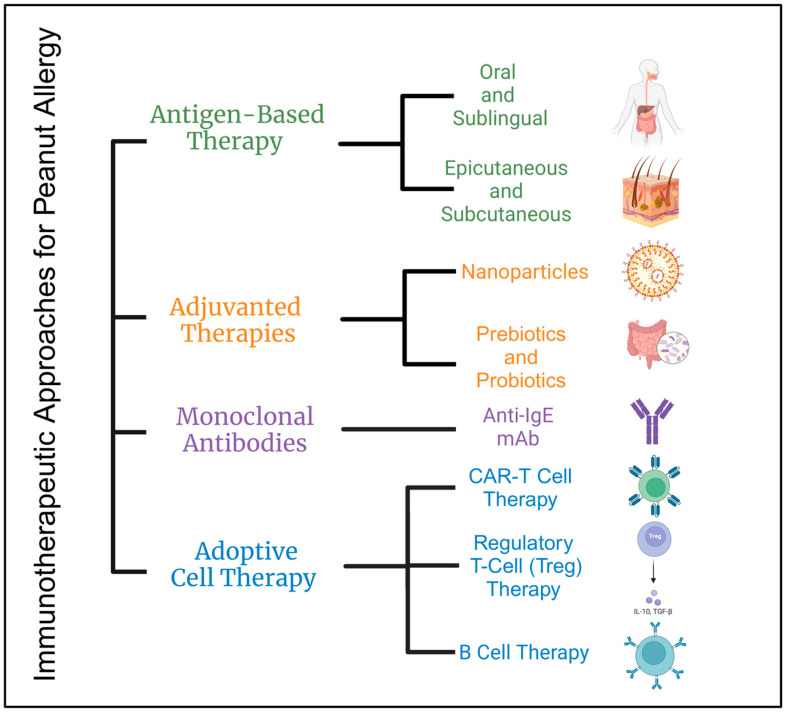
Overview of different immunotherapeutic approaches for peanut allergy: The figure above summarizes the various therapeutic approaches utilized for the treatment of peanut allergy.

Various treatment options for peanut allergy have been explored (Figure 2), each with unique benefits and risks. Subcutaneous immunotherapy (SCIT) poses a significant risk of severe reactions, while alternatives such as epicutaneous immunotherapy (EPIT), oral immunotherapy (OIT), and sublingual immunotherapy (SLIT) show promise in promoting immune tolerance and desensitization with safer profiles. Monoclonal antibodies like Omalizumab have proven effective in reducing IgE-mediated allergic reactions. Adjuvant therapies, which enhance immune responses using nanoparticles and probiotics, have shown potential in both preclinical and clinical settings. Additionally, adoptive cell therapies, including regulatory T cells (Tregs) and chimeric antigen receptor (CAR) T cells, offer innovative approaches to modulating immune responses and achieving long-term tolerance to peanut allergens. Collectively, these therapeutic strategies contribute to a comprehensive understanding of peanut allergy management, offering hope for safer and more effective treatments.

## Figures and Tables

**Table 1:** Comparison of antigen-specific immunotherapy (AIT) approaches for peanut allergy.

AIT Approach	Advantages	Limitations	Preferred Clinical Scenarios
SCIT (subcutaneous immunotherapy)	- Well established in airborne allergies - Potential for long-term tolerance with adjuvant use (e.g., alum)	- High risk of systemic anaphylaxis - Requires modification to reduce severe reactions - Limited success in peanut allergy trials	- Currently not widely used for food allergies due to safety concerns - May require modification for safer clinical application
EPIT (epicutaneous immunotherapy)	- Noninvasive - Demonstrated safety in pediatric peanut allergy trials - Lower risk of systemic reactions	- Limited desensitization compared to OIT - Requires prolonged treatment for effectiveness	- Suitable for pediatric patients or those at high risk of systemic reactions - Ideal for patients unable to tolerate OIT
OIT (oral immunotherapy)	- High rates of clinical desensitization - FDA approved (Palforzia) - Increases threshold for allergic reactions	- Risk of gastrointestinal side effects and anaphylaxis - Requires strict adherence and medical supervision	- Patients willing to undergo desensitization despite potential side effects - Recommended for children aged 4–17 (FDA-approved)
SLIT (sublingual immunotherapy)	- Safer that OIT with fewer systemic reactions - Effective at lower allergen doses	- Lower levels of desensitization compared to OIT - Requires long treatment duration	- Patients unable to tolerate OIT but still seeking desensitization - Those at moderate risk of anaphylaxis

This table provides a comparative overview of four antigen-specific immunotherapy approaches—subcutaneous immunotherapy (SCIT), epicutaneous immunotherapy (EPIT), oral immunotherapy (OIT), and sublingual immunotherapy (SLIT)—highlighting their advantages, limitations, and preferred clinical applications. Each approach varies in its efficacy, safety profile, and suitability for different patient populations, offering distinct benefits and challenges in peanut allergy treatment.

**Table 2:** Peanut allergy immunotherapy clinical trials reviewed.

Study	ID Number	Year	Arms	Results
Epicutaneous Immunotherapy in Peanut Allergy in Children (ARACHILD)	NCT01197053	2015	EPIT with Viaskin^®^ placebo or control	Completed, not recruiting, results posted
Peanut Oral Immunotherapy and Anti-Immunoglobulin E (IgE) for Peanut Allergy (PAIE/Xolair)	NCT00932282	2018	Peanut OIT in combination with Omalizumab	Completed, not recruiting, results posted
Sublingual Immunotherapy for Peanut Allergy and Induction of Tolerance (SLIT-TLC)	NCT01373242	2019	DBPCFC peanut SLIT	Completed, not recruiting, results posted
Evaluating the Safety and Efficacy of Oral Encapsulated Fecal Microbiota Transplant in Peanut Allergic Patients	NCT02960074	2019	Biological: fecal microbiota capsule	Completed, not recruiting, no results posted
Safety and Efficacy Study of Viaskin^®^ Peanut in Peanut-allergic Young Children 1–3 Years of Age (EPITOPE)	NCT03211247	2021	Viaskin^®^ 250 mcg Viaskin^®^ 100 mcg placebo	Completed, not recruiting, results submitted
Omalizumab as Monotherapy and as Adjunct Therapy to Multi-Allergen OIT in Food Allergic Participants (OUTMATCH)	NCT03881696	2023	Omalizumab Omalizumab in combination with multi-allergen OIT	Active, not recruiting, results posted
Peanut Oral Immunotherapy Study of Early Intervention for Desensitization (POSEIDON)	NCT03736447	2023	AR101 powder placebo powder	Completed, not recruiting, results posted
Analyzing High-Dose Probiotic Peanut Oral Immunotherapy (PPOIT) and High-Dose Peanut Oral Immunotherapy (OIT) Versus Low-Dose Peanut OIT for Peanut Allergy	NCT06297083	2024	HD PPOIT HD OIT LD OIT	Withdrawn, no results posted

This table breaks down the different clinical trials utilizing several of the previously mentioned immunotherapeutic approaches for the treatment of peanut allergy. The study aims, clinical trial status, and approval year are included in the above categorization of the clinical trials.

## Abbreviations

The following abbreviations are used in this manuscript:

PA	Peanut allergy
AIT	Antigen-specific immunotherapy
SCIT	Subcutaneous immunotherapy
EPIT	Epicutaneous immunotherapy
OIT	Oral immunotherapy
SLIT	Sublingual immunotherapy
Tregs	Regulatory T cells
IL-#	Interleukins
Bregs	Regulatory B cells
GALT	Gut-associated lymphoid tissue
APC	Antigen presenting cells
Ig	Immunoglobulin
mAb	Monoclonal antibody
PLGA	Poly(lactic-co-glycolic acid)
DC	Dendritic cells
PRR	Pattern recognition receptors
PAMPS	Pathogen-associated molecular patterns
PPOIT	Peanut and probiotic oral immunotherapy
ACT	Adoptive cell therapy
CAR	Chimeric antigen receptors
OVA	Ovalbumin
DBPCFC	Double-blind, placebo-controlled food challenge
HD PPOIT	High-dose peanut and probiotic oral immunotherapy
HD OIT	High-dose oral immunotherapy
LD OIT	Low-dose oral immunotherapy
FMT	Fecal microbiota transplantation

## References

[1] Al-Ahmed N., Alsowaidi S., Vadas P. (2008). Peanut Allergy: An Overview. Allergy Asthma Clin. Immunol..

[2] Abbas M., Moussa M., Akel H. (2023). Type I Hypersensitivity Reaction.

[3] Sutanto H. (2024). Mechanobiology of Type 1 hypersensitivity: Elucidating the impacts of mechanical forces in allergic reactions. Mechanobiol. Med..

[4] Lieberman J.A., Gupta R.S., Knibb R.C., Haselkorn T., Tilles S., Mack D.P., Pouessel G. (2020). The global burden of illness of peanut allergy: A comprehensive literature review. Allergy.

[5] Warren C., Lei D., Sicherer S., Schleimer R., Gupta R. (2021). Prevalence and characteristics of peanut allergy in US adults. J. Allergy Clin. Immunol..

[6] Cannon H.E. (2018). The Economic Impact of Peanut Allergies. Am. J. Manag. Care.

[7] Shah F., Shi A., Ashley J., Kronfel C., Wang Q., Maleki S.J., Adhikari B., Zhang J. (2019). Peanut Allergy: Characteristics and Approaches for Mitigation. Compr. Rev. Food Sci. Food Saf..

[8] Adytia G.J., Sutanto H., Pratiwi L., Fetarayani D. (2025). Advances in Synthetic Immunology for Targeted Treatment of Systemic Autoimmune Diseases: Opportuni-ties, Challenges, and Future Directions. Immuno.

[9] Bublin M., Breiteneder H. (2014). Developing Therapies for Peanut Allergy. Int. Arch. Allergy Immunol..

[10] Akdis C.A., Akdis M. (2015). Mechanisms of allergen-specific immunotherapy and immune tolerance to allergens. World Allergy Organ. J..

[11] Satitsuksanoa P., Jansen K., Głobińska A., van de Veen W., Akdis M. (2018). Regulatory Immune Mechanisms in Tolerance to Food Allergy. Front. Immunol..

[12] Quirosa E.B.D., Seoane-Reula E., Alonso-Lebrero E., Pion M., Correa-Rocha R. (2018). The role of regulatory T cells in the acquisition of tolerance to food allergens in children. Allergol. Immunopathol..

[13] Johnston C.J., Smyth D.J., Dresser D.W., Maizels R.M. (2016). TGF-β in tolerance, development and regulation of immunity. Cell. Immunol..

[14] Larionov A.S., Stavrakaki I., Kim E., Sicherer S.H., Wood R.A., Couroux P.R., Gonzalez-Reyes E.G., Hussain I., Tilles S.A., Opstelten D.-J. (2019). Development Of A Subcutaneous Immunotherapy (SCIT) With A Modified Peanut Extract Formulation For The Treatment Of Peanut Allergy. J. Allergy Clin. Immunol..

[15] Courey M.S., Pletcher S.D. (2016). 49–Upper Airway Disorders. Murray Nadel’s Textb. Respir. Med..

[16] Krishna S.S., Farhana S.A., TP A., Hussain S.M., Viswanad V., Nasr M.H., Sahu R.K., Khan J. (2023). Modulation of immune response by nanoparticle-based immunotherapy against food allergens. Front. Immunol..

[17] Gamazo C., D’Amelio C., Gastaminza G., Ferrer M., Irache J.M. (2017). Adjuvants for allergy immunotherapeutics. Hum. Vaccines Immunother..

[18] Hervé P.-L., Dioszeghy V., Matthews K., Bee K.J., Campbell D.E., Sampson H.A. (2023). Recent advances in epicutaneous immunotherapy and potential applications in food allergy. Front. Allergy.

[19] Mondoulet L., Dioszeghy V., Vanoirbeek J.A., Nemery B., Dupont C., Benhamou P.-H. (2010). Epicutaneous Immunotherapy Using a New Epicutaneous Delivery System in Mice Sensitized to Peanuts. Int. Arch. Allergy Immunol..

[20] Pongracic J.A., Gagnon R., Sussman G., Siri D., Oriel R.C., Brown-Whitehorn T.F., Green T.D., Campbell D.E., Anvari S., Berger W.E. (2022). Safety of Epicutaneous Immunotherapy in Peanut-Allergic Children: REALISE Randomized Clinical Trial Results. J. Allergy Clin. Immunol. Pr..

[21] Jones S.M., Sicherer S.H., Burks A.W., Leung D.Y., Lindblad R.W., Dawson P., Henning A.K., Berin M.C., Chiang D., Vickery B.P. (2016). Epicutaneous immunotherapy for the treatment of peanut allergy in children and young adults. J. Allergy Clin. Immunol..

[22] Sampson H.A., Shreffler W.G., Yang W.H., Sussman G.L., Brown-Whitehorn T.F., Nadeau K.C., Cheema A.S., Leonard S.A., Pongracic J.A., Sauvage-Delebarre C. (2017). Effect of Varying Doses of Epicutaneous Immunotherapy vs Placebo on Reaction to Peanut Protein Ex-posure Among Patients With Peanut Sensitivity: A Randomized Clinical Trial. JAMA.

[23] Fleischer D.M., Greenhawt M., Sussman G., Bégin P., Nowak-Wegrzyn A., Petroni D., Beyer K., Brown-Whitehorn T., Hebert J., O’B Hourihane J. (2019). Effect of Epicutaneous Immunotherapy vs Placebo on Reaction to Peanut Protein Ingestion Among Children With Peanut Allergy: The PEPITES Randomized Clinical Trial. JAMA.

[24] Chow T.G., Parrish C., Bird J.A. (2020). Food allergy: Epicutaneous immunotherapy. J. Food Allergy.

[25] Liu E.G., Yin X., Swaminathan A., Eisenbarth S.C. (2021). Antigen-Presenting Cells in Food Tolerance and Allergy. Front. Immunol..

[26] Barshow S.M., Kulis M.D., Burks A.W., Kim E.H. (2021). Mechansims of Oral Immunotherapy. Clin. Exp. Allergy.

[27] Erlich D. (2022). Peanut Allergen Powder (Palforzia) for Peanut Allergy. Am. Fam. Physician.

[28] Anagostou K., Islam S., King Y., Foley L., Pasea L., Bond S., Palmer C., Deighton J., Ewan P., Clark A. (2014). Assessing the efficacy of oral immunotherapy for the densensitization of peanut allergy in children (STOP II): A phase 2 randomised controlled trial. Lancet.

[29] Borne G.E., Daniel C.P., Wagner M.J., Plaisance C.J., Nolen A., Kelkar R.A., Ahmadzadeh S., Myrcik D., Shekoohi S., Kaye A.D. (2023). Palforzia for Peanut Allergy: A Narrative Review and Update on a Novel Immunotherapy. Cureus.

[30] A Schworer S., Kim E.H. (2020). Sublingual Immunotherapy for Food Allergy and its Future Directions. Immunotherapy.

[31] Kim E.H., Keet C.A., Virkud Y.V., Chin S., Ye P., Penumarti A., Smeekens J., Guo R., Yue X., Li Q. (2023). Open-label study of the efficacy, safety, and durability of peanut sublingual immunotherapy in peanut-allergic children. J. Allergy Clin. Immunol..

[32] MacGinnitie A.J., Rachid R., Gragg H., Little S.V., Lakin P., Cianferoni A., Heimall J., Makhija M., Robison R., Chinthrajah R.S. (2016). Omalizumab facilitates rapid oral desensitization for peanut allergy. J. Allergy Clin. Immunol..

[33] Breedveld F.C. (2000). Therapeutic monoclonal antibodies. Lancet.

[34] Leung D.Y., Sampson H.A., Yunginger J.W., Burks A.W.J., Schneider L.C., Wortel C.H., Davis F.M., Hyun J.D., Shanahan W.R.J. (2003). Effect of Anti-IgE Therapy in Patients with Peanut Allergy. N. Engl. J. Med..

[35] Wood R.A., Togias A., Sicherer S.H., Shreffler W.G., Kim E.H., Jones S.M., Leung D.Y., Vickery B.P., Bird J.A., Spergel J.M. (2024). Omalizumab for the Treatment of Multiple Food Allergies. N. Engl. J. Med..

[36] Johnson-Weaver B.T., Staats H.F., Burks A.W., Kulis M.D. (2018). Adjuvanted Immunotherapy Approaches for Peanut Allergy. Front. Immunol..

[37] Beilvert F., Tissot A., Langelot M., Mével M., Chatin B., Lair D., Magnan A., Pitard B. (2012). DNA/Amphiphilic Block Copolymer Nanospheres Reduce Asthmatic Response in a Mouse Model of Allergic Asthma. Hum. Gene Ther..

[38] Srivastava K.D., Siefert A., Fahmy T.M., Caplan M.J., Li X.-M., Sampson H.A. (2016). Investigation of peanut oral immunotherapy with CpG/peanut nanoparticles in a murine model of peanut allergy. J. Allergy Clin. Immunol..

[39] Rebouças J.D.S., Irache J.M., Camacho A.I., Gastaminza G., Sanz M.L., Ferrer M., Gamazo C. (2014). Immunogenicity of Peanut Proteins Containing Poly(Anhydride) Nanoparticles. Clin. Vaccine Immunol..

[40] Berin M.C., Shreffler W.G. (2008). TH2 adjuvants: Implications for food allergy. J. Allergy Clin. Immunol..

[41] Méndez J.L., Palomares F., Gómez F., Ramírez-López P., Ramos-Soriano J., Torres M.J., Mayorga C., Rojo J. (2021). Immunomodulatory Response of Toll-like Receptor Ligand–Peptide Conjugates in Food Allergy. ACS Chem. Biol..

[42] Wang X., Zhang P., Zhang X. (2021). Probiotics Regulate Gut Microbiota: An Effective Method to Improve Immunity. Molecules.

[43] Berni Canani R., Paparo L., Nocerino R., Di Scala C., Della Gatta G., Maddalena Y., Buono A., Bruno C., Voto L., Ercolini D. (2019). Gut Microbiome as Target for Innovative Strategies Against Food Allergy. Front. Immunol..

[44] Hsiao K.-C., Ponsonby A.-L., Axelrad C., Pitkin S., Tang M.L.K., Burks W., Donath S., Orsini F., Tey D., Robinson M. (2017). Long-term clinical and immunological effects of probiotic and peanut oral immunotherapy after treatment cessation: 4-year follow-up of a randomised, double-blind, placebo-controlled trial. Lancet Child Adolesc. Health.

[45] Tang M.L.K., Ponsonby A.-L., Orsini F., Tey D., Robinson M., Su E.L., Licciardi P., Burks W., Donath S. (2015). Administration of a probiotic with peanut oral immunotherapy: A randomized trial. J. Allergy Clin. Immunol..

[46] Kverneland A.H., Chamberlain C.A., Borch T.H., Nielsen M., Mørk S.K., Kjeldsen J.W., Lorentzen C.L., Jørgensen L.P., Riis L.B., Yde C.W. (2021). Adoptive cell therapy with tumor-infiltrating lymphocytes supported by checkpoint inhibition across multiple solid cancer types. J. Immunother. Cancer.

[47] Bellinghausen I., Khatri R., Saloga J. (2022). Current Strategies to Modulate Regulatory T Cell Activity in Allergic Inflammation. Front. Immunol..

[48] Raffin C., Vo L.T., Bluestone J.A. (2020). Treg cell-based therapies: Challenges and perspectives. Nat. Rev. Immunol..

[49] Tang M.L.K., Mullins R.J. (2017). Food allergy: Is prevalence increasing?. Int. Med. J..

[50] Marco R.C.D., Monzo H.J., Ojala P.M. (2023). CAR T Cell Therapy: A Versatile Living Drug. Int. J. Mol. Sci..

[51] Ward D.E., Fay B.L., Adejuwon A., Han H., Ma Z. (2018). Chimeric Antigen Receptors Based on Low Affinity Mutants of FcεRI Re-direct T Cell Specificity to Cells Expressing Membrane IgE. Front. Immunol..

[52] Abdeladhim M., Zhang A.-H., Kropp L.E., Lindrose A.R., Venkatesha S.H., Mitre E., Scott D.W. (2019). Engineered ovalbumin-expressing regulatory T cells protect against anaphylaxis in ovalbumin-sensitized mice. Clin. Immunol..

[53] Prickler L., Baranyi U., Mengrelis K., Weijler A.M., Kainz V., Kratzer B., Steiner R., Mucha J., Rudoph E., Pilat N. (2023). Adoptive transfer of allergen-expressing B cells prevents IgE-mediated allergy. Front. Immunol..

[54] Chernikova D.A., Zhao M.Y., Jacobs J.P. (2022). Microbiome Therapeutics for Food Allergy. Nutrients.

